# Genetic variants as predictors of toxicity and response in patients with non‐small cell lung cancer undergoing first‐line platinum‐based chemotherapy: Design of the multicenter PGxLUNG study

**DOI:** 10.1111/1759-7714.13683

**Published:** 2020-10-19

**Authors:** Corine de Jong, Gerarda J.M. Herder, Vera H.M. Deneer

**Affiliations:** ^1^ Department of Clinical Pharmacy St. Antonius Hospital Nieuwegein/Utrecht The Netherlands; ^2^ Department of Clinical Pharmacy, Division of Laboratories, Pharmacy and Biomedical Genetics University Medical Center Utrecht Utrecht The Netherlands; ^3^ Department of Pulmonology Meander Medical Center Amersfoort The Netherlands; ^4^ Division of Pharmacoepidemiology and Clinical Pharmacology, Utrecht Institute for Pharmaceutical Sciences Utrecht University Utrecht The Netherlands

**Keywords:** carboplatin; cisplatine; chemotherapy‐induced toxicity, genome‐wide association study (GWAS), health‐related quality of life (HRQOL);non‐small cell lung cancer (NSCLC); skeletal muscle mass (SMM); Pharmacogenetics; platinum‐based chemotherapy

## Abstract

**Introduction:**

Platinum–based chemotherapy is currently the most frequently applied first‐line treatment for patients with advanced non‐small cell lung cancer (NSCLC) without targetable mutations or high PD‐L1 expression. Unfortunately, chemotherapy‐induced toxicity is prevalent and may affect patients' quality of life to a considerable extent. Presumably, genetic variants of genes, coding for proteins involved in the processes of the development of toxicity, may be of interest as predictors of benefits and harms of platinum‐based chemotherapy.

The primary objective of the study is to investigate the influence of genetic variants on the incidence of chemotherapy‐induced toxicity in patients with NSCLC undergoing first‐line platinum‐based chemotherapy. The main secondary objectives are to study the association between genetic variants and treatment response and to study the association between skeletal muscle mass (SMM) as well as patient‐reported health‐related quality of life (HRQOL) and treatment response and toxicity.

**Methods:**

In this multicenter prospective follow‐up study, a total of 350 patients with NSCLC (stage II–IV) undergoing first‐line platinum‐based chemotherapy will be included. Blood samples for DNA isolation and genotyping, questionnaires and data on patients risk factors and disease stage will be recorded. The primary endpoint is chemotherapy‐induced (non‐)hematological toxicity, comprising; nephrotoxicity, neuropathy, esophagitis, ototoxicity, pneumonitis, gastrointestinal toxicity, anemia, leukocytopenia, neutropenia and thrombocytopenia. Secondary endpoints include dose‐limiting toxicity, HRQOL, and treatment response (radiological response [RECIST 1.1] and overall survival [OS]).

**Discussion:**

Results of the PGxLUNG study will be primarily used to determine the influence of genetic variants on the incidence of chemotherapy‐induced toxicity in patients with NSCLC undergoing first‐line platinum‐based chemotherapy.

## Introduction

Lung cancer remains the leading cause of cancer‐related death worldwide, in which non‐small cell lung cancer (NSCLC) accounts for nearly 85% of all cases.^1^ For decades, therapeutic treatment of NSCLC consisted of platinum‐based chemotherapy, which has been shown to be moderately effective on progression‐free and overall survival.^2^, ^3^ However, identification of targetable mutations (eg, an epidermal growth factor receptor [*EGFR*] mutation or anaplastic lymphoma kinase [*ALK*] rearrangement) have led to changes in treatment options over the past few years.^4^, ^5^ In addition, the introduction of immunotherapy has recently led to new treatment perspectives and strategies. Even though there are promising changes in treatment options for NSCLC, only a minority of patients will benefit from these new first‐line therapies. In addition, platinum‐based chemotherapy is also given as first‐line treatment in combination with immunotherapy, or as second‐line treatment after targeted therapy.^6^, ^7^, ^8^, ^9^ Therefore, although there are rapid transformations in the therapeutic landscape, nowadays platinum–based chemotherapy remains the mainstay for treatment of NSCLC patients worldwide.

Unfortunately, chemotherapy‐induced toxicity is prevalent (20%–30%) and may affect patients' quality of life to a considerable extent.^10^, ^11^ Chemotherapy is frequently part of palliative care, and it is therefore of the utmost importance to prevent treatment complications. However, identifying patients who are at high risk of developing serious adverse events is difficult, since predictive tools are lacking. Genetic variants of genes, coding for proteins involved in the processes of development of toxicity, may be of interest as predictors of benefits and harms. Previous studies in patients with different kinds of malignancies report genetic variants in organic transporter molecules genes (*OCT2*), DNA repair enzyme genes (*ERCC1*, *ERCC2*), genes encoding tumor suppressor proteins (TP53), or metabolic enzymes involved in platinum detoxification (*GST1*) and other pharmacodynamic genes (*COMT*) among others, may be involved in the development of toxicity.^12^, ^13^, ^14^ Other possible prognostic and predictive parameters for treatment response and toxicity are based upon body composition. This could be of relevance since changes in body composition in patients with cancer are prevalent due to cachexia‐associated muscle mass loss.^15^ Moreover, low lean body weight, and skeletal muscle mass (SMM) depletion (sarcopenia), together with the radiodensity of skeletal muscle tissue, have been suggested to be associated with a higher incidence of chemotherapy‐induced toxicity in cancer patients.^15^, ^16^, ^17^, ^18^, ^19^

Hence, currently, little is known about the possible associations between genetic variants as well as skeletal muscle depletion and platinum‐based chemotherapy‐induced toxicity in patients with NSCLC.

## Objectives

The primary objective of the Pharmacogenetics Lung Cancer (PGxLUNG) study is to investigate the influence of genetic variants on the incidence of chemotherapy‐induced toxicity in patients with NSCLC undergoing first‐line platinum‐based chemotherapy in a multicenter prospective follow‐up study.

The main secondary objectives are to study the association between genetic variants and treatment response, to study the association between skeletal muscle mass (SMM) as well as patient‐reported health‐related quality of life (HRQOL) and treatment response and toxicity.

## Methods/design

### Setting

This study is a prospective follow‐up study with a multicenter design, conducted in one academic hospital (University Medical Center Utrecht), two teaching hospitals (St. Antonius Hospital Nieuwegein/Utrecht, Meander Medical Center Amersfoort) and three general hospitals (Diakonessenhuis Utrecht/Zeist, Groene Hart Ziekenhuis Gouda, Ziekenhuis Rivierenland Tiel), all in the Netherlands.

### Eligibility

The study population consists of NSCLC patients (stage II–IV) undergoing first‐line platinum‐based chemotherapy as part of routine patient care.

Inclusion criteria: (i) Older than 18 years of age; (ii) radiologically‐confirmed NSCLC (stage II–IV); and (iii) first‐line treatment with platinum‐based (cisplatin or carboplatin) chemotherapy or chemoradiotherapy (according to the contemporary ESMO Clinical Practice Guidelines).^4^, ^5^ Patients are platinum‐based chemotherapy‐naïve and treatment is planned or has been initiated.

Exclusion criteria: (i) Cognitive impairment; and (ii) unable to read and write Dutch.

All patients receive at least one cycle of a platinum‐agent combined with a chemotherapeutic agent (eg, etoposide, gemcitabine, pemetrexed, paclitaxel), targeted therapy (bevacizumab) and/or immunotherapy (eg, atezolizumab, nivolumab, pembrolizumab), depending on tumor histology and patient characteristics. Radiotherapy can be either sequential or concurrent, according to the physician's choice. Patients can enroll in the study prior to initiation of chemotherapy or after chemotherapy has been initiated. All treatment procedures (ie, diagnostic work‐up, laboratory tests) will be according to local clinical practice for routine patient care. The end of study is the date of the end of follow‐up of the last included patient.

### Ethical considerations

The protocol complied with the Good Clinical Practice guidelines and the Declaration of Helsinki (64th WMA General Assembly, Fortaleza, Brazil, October 2013), and was approved by the accredited Medical Research Ethics Committee in Nieuwegein (MEC‐U, number R15.056). The study was registered in The Netherlands National Trial Register (NTR) on 26 April 2016 (NTR number NL5373610015). The treating medical doctor will obtain written informed consent from each participant.

### Measurements

#### Blood sampling

An EDTA‐blood sample for genotyping will be collected in all patients. For patients who enroll in the study prior to initiation of chemotherapy extra EDTA‐blood and serum samples will be collected for measurement of biomarkers possibly associated with treatment response and/or toxicity at four points in time (Table 1). Serum and plasma samples will be processed and stored at –80°C until further analysis. The samples will be coded and stored for a period of 30 years, which provides the opportunity to perform additional research in the future.

**Table 1:** Schedule of measurements and data collection

Measurements/variables	Prior to cycle 1 Week 0	Prior to cycle 2 Week 3	Prior to cycle 3 Week 6	Prior to cycle 4 Week 9	Follow‐up 3 months	Follow‐up 6 months	Follow‐up 12 months
Blood sampling	X				X^†^	X^†^	X^†^
HRQOL assessment	X^†^				X^†^	X^†^	X^†^
Patient demographics	X						
Disease characteristics	X						
Clinical observations	X	X	X	X	X	X	X
Treatment characteristics	X	X	X	X			
Biochemical characteristics	X	X	X	X	X	X	X
Chemotherapy‐induced toxicity		X	X	X	X	X	X
Radiological response			X		X	X	X
Survival status							X

^†^ For patients who enroll in the study prior to initiation of chemotherapy.

HRQOL, health‐related quality of life.

#### Sample processing and genotyping

DNA samples will be obtained from EDTA‐blood samples using the EZ1 DNA Blood 200 μl kit (Qiagen, Hilden, Germany). DNA isolation will be performed according to validated in‐house protocols of the Pharmacogenetics, Pharmaceutical and Toxicological Laboratory (FarmaToxLab) of the Department of Clinical Pharmacy (ISO15189 certified), St. Antonius Hospital Nieuwegein/Utrecht. Single nucleotide polymorphisms (SNPs) will be genotyped by using Kompetitive allele specific PCR (KASP) at LGC Genomics (Hoddesdon, UK) and by using the Infinium Global Screening Array‐24 Kit (Illumina, San Diego, CA) at Life and Brain (Bonn, Germany).

#### Health‐related quality of life

Patients who enroll in the study prior to initiation of chemotherapy will be asked to complete questionnaires regarding health‐related quality of life (HRQOL) at treatment initiation and, three, six and 12 months after starting chemotherapy (Table 1). The first hardcopy questionnaire will be handed over by a research nurse. Follow‐up questionnaires will be sent as a hardcopy to the patient's home address by the research nurse. To assess HRQOL four instruments will be used; EQ‐5D, EORTC QLQ‐C30, EORTC QLQ‐LC13 and EORTC QLQ‐CIPN20 (Table S1). All questionnaires are widely‐used and internationally validated.^20^, ^21^, ^22^, ^23^

### Endpoints

The primary endpoint is chemotherapy‐induced toxicity. Chemotherapy‐induced toxicity is defined as hematological and nonhematological toxicity. Nonhematological toxicity comprised nephrotoxicity, neuropathy, esophagitis and pneumonitis. Hematological toxicity includes anemia, leukocytopenia, neutropenia and thrombocytopenia. Chemotherapy‐induced toxicity will be assessed using the contemporary Common Terminology Criteria for Adverse Events (CTC‐AE) (version 4.03 or higher) or predefined definitions (Table S2).^24^

Secondary endpoints comprise SMM, patient‐reported HRQOL (Table S1), dose‐limiting toxicity defined as “switching treatment” (cisplatin to carboplatin), “treatment delay” (≥seven days from initially planned), treatment de‐escalation (dose reduction ≥25% of chemotherapeutic agent), early treatment termination and treatment‐related hospital admissions (days of hospitalization) (Table S3), changes in biochemical characteristics, biomarker levels and hematological parameters, treatment response in terms of radiological response (according to the World Health Organization (WHO) Response Evaluation Criteria in Solid Tumors (RECIST 1.1)), ^25^ and overall survival (OS).

### Data collection

A data management plan, comprising detailed information about data collection, managing and storing of research data has been developed. Clinical data will be extracted from the hospital's electronic information systems and managed using web‐based REDCap electronic data capture tools.^26^ Beforehand, ranges will be defined in the file for all data values to ensure data validity and integrity. To reduce interobserver variability in gathering and entering data, only four trained individuals will be involved in the data collection process. Data collection will stop one year after start of first‐line platinum‐based chemotherapy. Patient data from this study will be coded. Only coded data will be analyzed and the results will be published anonymously.

The following parameters and endpoints, of which some are considered to be potentially confounding variables, at baseline and at six follow‐up time points as shown in Table 1, will be collected:

- *Patient demographics*: Age at diagnosis, gender, ethnicity, smoking status, alcohol (ab)use.
- *Clinical observations*: Charlson comorbidity index,^27^ Eastern Cooperative Oncology Group Performance status (ECOG PS),^28^ anthropometric measurements (weight, length, body mass index [BMI]), skeletal muscle measurements by pretreatment and follow‐up imaging (using fluorine‐18 deoxyglucose positron emission tomography [FDG‐PET‐] computed tomography [CT] scans as part of standard clinical care).
- *Disease characteristics*: Disease stage (according to the contemporary TNM Classification of Malignant Tumors, seventh edition or higher),^29^, ^30^ histological tumor subtype, manifestation of metastases in the central nervous system.
- *Treatment characteristics*: Platinum‐based agent, dosage, number of cycles, radiotherapy.
- *Biochemical characteristics and biomarker levels*: Serum creatinine, urea, albumin, magnesium, calcium, lactate dehydrogenase (LDH), carcinoembryonic antigen (CEA), cancer antigen 125 (CA 125).
- *Chemotherapy‐induced toxicity*: Nonhematological toxicity (nephrotoxicity (estimated glomerular filtration rate (eGFR, according to CKD‐EPI), serum creatinine),^31^, ^32^ neuropathy, esophagitis, ototoxicity, pneumonitis, gastrointestinal toxicity) and hematological toxicity (anemia (Hb level), leukocytopenia (leukocyte count), neutropenia (neutrophils count), thrombocytopenia (platelet count)).
- *Treatment response*: Radiological response and survival status. Radiological response will be measured after two and four chemotherapy cycles (at six and 12 weeks after treatment initiation, respectively) by CT, FDG‐PET and/or magnetic resonance imaging (MRI), as part of standard clinical care. Radiological response will be categorized as progressive disease (PD), stable disease (SD), partial response (PR) or complete response (CR), according to RECIST 1.1.^25^

### Sample size considerations

The sample size calculation is based on a candidate gene approach and on the assumption that approximately 30% of the patients undergoing platinum‐based chemotherapy will develop chemotherapy‐induced toxicity.^10^, ^11^ Common genetic variants will be selected. For example, a genotype or allele frequency of 0.05, 30% of patients with toxicity and a total of 333 patients, implies a detection of true odds ratios (OR) for toxicity of 0.43 or 2.03 in subjects with the genotype or allele of interest relative to subjects without this genotype or allele with a power of 0.8 and a type I error probability of 0.05. Since genetic testing can fail in 3%–5% of the cases, the total number of patients needed in this study is 350.

### Data analysis

Standard statistical analysis will be performed by using SPSS version 25.0 or higher (IBM SPSS Statistics) and GraphPad Prism version 8.3 or higher. Standard summary statistics will be used to describe the sample data set. Categorical data will be expressed as frequencies and percentages. Continuous variables will be expressed as mean ± SD or median (ranges). Categorical data will be compared between groups by using the chi‐square test and continuous data by Student's *t*‐test or ANOVA when appropriate. In the primary analysis, toxicity will be defined as CTC‐AE ≥grade 1. Depending on the incidence of toxicity grade 2 or higher for the individual endpoints, further stratification will be carried out.

To examine the association between genetic variants and the risk for development of chemotherapy‐induced toxicity, different approaches will be used. A candidate gene approach will be used and genome‐wide association studies (GWAS) will be performed. Within the candidate gene approach, logistic regression models will be used to test for associations between genetic variants and toxicity expressed as categorical variables and odds ratios (OR) with 95% confidence intervals (CI) will be calculated. If a genetic association is found, correcting for multiple testing will be performed by using the false discovery rate test (q value threshold 0.20).^33^ GWAS and quality control will be performed using PLINK version 1.9 or higher. Standard quality control (ie, by filtering on SNP call rate, Hardy‐Weinberg equilibrium, minor allele frequency (MAF) and population stratification (with commonly accepted thresholds based on current literature)) pre‐ and post‐genotype imputation will be applied.^34^ Imputation will be conducted on the University of Michigan Imputation Server.^35^ To correct for multiple comparisons, conventional methods such as Bonferroni correction (ie, *P* ≤ 5·10^−8^ and *P* ≤ 5·10^−5^ for genome‐wide significance and near‐significance (suggestive) association respectively) will be used to conduct these analyses.

Genetic variants will also be studied for association with radiological response (according to RECIST 1.1)^25^ and OS. Individual patient overall survival time will be defined as the time difference between the date of treatment initiation until death. For patients who are alive by the end of follow‐up (12 months after chemotherapy initiation) data will be censored. Median overall survival will be plotted in Kaplan‐Meier curves and groups will be compared by using the log rank test. Hazard ratios (HR) with 95% CI will be calculated with Cox proportional hazard modeling. The multivariate setting of both logistic regression and Cox proportional hazard regression will be used to take potential confounding variables, specifically for the endpoint in question, into account and to calculate adjusted OR (ORadj) and adjusted HR (HRadj). In addition, when appropriate, stratification analysis (eg, based on platinum‐based agent, histological tumor subtype or use of additional radiotherapy) will be performed.

For the analysis of the secondary endpoints, the statistical methods as described above will be used, when appropriate. In addition, univariate and multivariate linear regression analysis will be performed, when indicated.

## Discussion

The results of this prospective follow‐up study with a multicenter design will be used to determine the influence of genetic variants on the incidence of chemotherapy‐induced toxicity in patients with NSCLC undergoing first‐line platinum‐based chemotherapy. In addition, the association between genetic variants and treatment response, the association between SMM as well as patient‐reported HRQOL with treatment response and toxicity will be assessed. Using a personalized medicine approach, the results may be used in the individualization of therapy based on the patient's clinical risk factors and genotype. Results of the PGxLUNG study may translate into minimisation of harm and contribute to improvement of quality of life of patients with NSCLC undergoing platinum‐based chemotherapy, which is still the treatment of first choice for the majority of NSCLC patients worldwide.

### Trial status

Patient recruitment and inclusion took place between February 2016 and August 2019. Follow‐up and data collection is ongoing.

## Disclosure

The authors have no conflicts of interest to declare.

## Supporting information


**Table S1.** Patient‐reported outcomes
**Table S2.** Chemotherapy‐induced toxicity
**Table S3.** Dose‐limiting toxicity
