# Supplementary material for: Genetic variants as predictors of toxicity and response in patients with non‐small cell lung cancer undergoing first‐line platinum‐based chemotherapy: Design of the multicenter PGxLUNG study
Source: Thorac Cancer. 2020 Oct 19;11(12):3634–40. doi: 10.1111/1759-7714.13683 (PMC7705630; doi:10.1111/1759-7714.13683)
Supplement: Supplementary file 1 — Table S1. Patient‐reported outcomes Table S2. Chemotherapy‐induced toxicity Table S3. Dose‐limiting toxicity [file TCA-11-3634-s001.docx]

**SUPPLEMENTARY APPENDIX**

**Table S1 *Patient-reported outcomes***

| Questionnaire | Aim of the questionnaire |
| --- | --- |
| EQ-5D  EQ VAS | European Quality of Life five dimension questionnaire (EQ-5D) is used to assesses health-related quality of life (HRQOL) in five socially relevant domains; mobility, self-care, usual activities, pain or discomfort and anxiety or depression.  The EQ-5D questionnaire is accompanied by a Visual Analogue Scale (EQ VAS) on which the subject is asked to provide a self-assessment of their own health in a range from 0 (worst imaginable health state) to 100 (best imaginable health state). |
| EORTC QLQ-C30  Version 3.0 | European Organisation for Research and Treatment of Cancer Quality of Life Questionnaire (EORTC QLQ-C30) is a questionnaire developed to assess the quality of life of cancer patients. |
| EORTC QLQ-LC13 | European Organisation for Research and Treatment of Cancer Quality of Life Questionnaire – Lung cancer (EORTC QLQ-LC13) is a questionnaire developed to assess the quality of life of lung cancer patients. It is a disease-specific supplement of the EORTC QLQ-C30 questionnaire. |
| EORTC QLQ-CIPN20 | European Organisation for Research and Treatment of Cancer Quality of Life Questionnaire - Chemotherapy-induced peripheral neuropathy (EORTC QLQ-CIPN20) is a questionnaire developed to assess the quality of life of cancer patients specified on peripheral neuropathy as a frequently occurring side-effect of chemotherapy. |

**Table S2 *Chemotherapy-induced toxicity***

| Category | Toxicity | Definition |
| --- | --- | --- |
| Nonhematological | **Esophagitis** | A disorder characterized by inflammation of the esophageal wall.  Grade 1: Asymptomatic; clinical or diagnostic observations only; intervention not indicated.  Grade 2: Symptomatic; altered eating/swallowing; oral supplements indicated.  Grade 3: Severely altered eating/swallowing; tube feeding, TPN, or hospitalization indicated.  Grade 4: Life-threatening consequences; urgent operative intervention indicated.  Grade 5: Death. |
|  | **Nephrotoxicity** | Acute kidney injury: A disorder characterized by the acute loss of renal function and is traditionally classified as pre-renal (low blood flow into kidney), renal (kidney damage) and post renal causes (ureteral or bladder outflow obstruction).  Grade 1: Creatinine level increase of >0.3 mg/dL (≈26 µmol/L); creatinine 1.5 -2.0 x above baseline.  Grade 2: Creatinine 2 to 3 times above baseline.  Grade 3: Creatinine >3 times baseline or >4.0 mg/dL (≈353 µmol/L); hospitalization indicated.  Grade 4: Life-threatening consequences; dialysis indicated.  Grade 5: Death. |
|  | **Ototoxicity** | Grade 1: No symptoms of hearing loss.  Grade 2: Hearing loss, no intervention.  Grade 3: Hearing loss, requiring intervention.  Grade 4: Profound bilateral hearing loss.  Grade 5: - |
|  | **Peripheral sensory neuropathy** | A disorder characterized by damage or dysfunction of the peripheral sensory nerves.  Grade 1: Asymptomatic; loss of deep tendon reflexes or paresthesia.  Grade 2: Moderate symptoms; limiting instrumental ADL.  Grade 3: Severe symptoms; limiting self-care ADL.  Grade 4: Life-threatening consequences; urgent intervention indicated.  Grade 5: Death. |
|  | **Pneumonitis** | A disorder characterized by inflammation focally or diffusely affecting the lung parenchyma.  Grade 1: Asymptomatic; clinical or diagnostic observations only; intervention not indicated.  Grade 2: Symptomatic; medical intervention indicated; limiting instrumental ADL.  Grade 3: Severe symptoms; limiting self-care ADL; oxygen indicated.  Grade 4: Life-threatening respiratory compromise; urgent intervention indicated (e.g., tracheotomy or intubation).  Grade 5: Death. |
|  | **Diarrhea** | A disorder characterized by an increase in frequency and/or loose watery bowel movements.  Grade 1: Increase of <4 stools per day over baseline.  Grade 2: Increase of 4-6 stools per day over baseline.  Grade 3: Increase of ≥7 stools per day over baseline; incontinence, hospitalization indicated, limiting self-care ADL.  Grade 4: Life-threatening consequences; urgent intervention indicated.  Grade 5: Death. |
|  | **Nausea** | A disorder characterized by a queasy sensation and/or the urge to vomit.  Grade 1: Loss of appetite without alteration in eating habits.  Grade 2: Oral intake decreased without significant weight loss, dehydration or malnutrition.  Grade 3: Inadequate oral caloric or fluid intake; tube feeding, TPN, or hospitalization indicated.  Grade 4: -  Grade 5: - |
|  | **Vomiting** | A disorder characterized by the reflexive act of ejecting the contents of the stomach through the mouth.  Grade 1: 1-2 episodes (separated by 5 minutes) in 24 hrs.  Grade 2: 3-5 episodes (separated by 5 minutes) in 24 hrs.  Grade 3: ≥6 episodes (separated by 5 minutes) in 24 hrs; tube feeding, TPN or hospitalization indicated.  Grade 4: Life-threatening consequences; urgent intervention indicated.  Grade 5: Death. |
| Hematological | **Anemia** | A disorder characterized by a reduction in the amount of hemoglobin (Hb) in blood. Signs and symptoms of anemia may include pallor of the skin and mucous membranes, shortness of breath, palpitations of the heart, soft systolic murmurs, lethargy, and fatigability.  Grade 1: Hb <LLN-6.2 mmol/L.  Grade 2: Hb <6.2 -4.9 mmol/L.  Grade 3: Hb <4.9 mmol/L, transfusion indicated.  Grade 4: Life-threatening consequences; urgent intervention indicated.  Grade 5: Death. |
|  | **Neutropenia** | A finding based on laboratory test results that indicate a decrease in number of neutrophils in a blood specimen.  Grade 1: neutrophils <LLN-1.5·10^9^/L.  Grade 2: neutrophils <1.5-1.0·10^9^/L.  Grade 3: neutrophils <1.0-0.5·10^9^/L.  Grade 4: neutrophils <0.5·10^9^/L.  Grade 5: - |
|  | **Platelet count decreased** | A finding based on laboratory test results that indicate a decrease in number of platelets in a blood specimen.  Grade 1: Platelet count <LLN -75·10^9^/L.  Grade 2: Platelet count <75·10^9^/L - 50·10^9^/L.  Grade 3: Platelet count <50·10^9^/L - 25·10^9^/L.  Grade 4: Platelet count <25·10^9^/L.  Grade 5: - |

Abbreviations: ADL: Activities of daily living. Instrumental ADL refer to preparing meals, shopping for groceries or clothes, using the telephone, managing money, etc. Self-care ADL refer to bathing, dressing and undressing, feeding self, using the toilet, taking medications, and not bedridden. Hb: Hemoglobin. LLN: Lower limit of normal. TPN: total parenteral nutrition.

**Table S3 *Dose-limiting toxicity***

| Toxicity | Definition |
| --- | --- |
| Treatment delay | The intended treatment as stated in the medical record is later started or resumed. The reason for rescheduling will be categorized in the database (e.g. due to chemotherapy-induced toxicity, disease progression, at patients request, other reasons). Treatment delay will be defined as treatment ≥7 days later than initially planned. |
| Treatment de-escalation | The intended treatment as stated in the medical record is not given according to the standard treatment protocol (e.g. dose reduction ≥25% or early treatment termination). The reason for treatment de-escalation will be categorized in the database (e.g. due to chemotherapy-induced toxicity, disease progression, at patients request, other reasons). |
| Switching treatment | The intended treatment as stated in the medical record is not completed, however the treatment is not discontinued but changed (e.g. cisplatin switched to carboplatin). The reason for switching treatment will be categorized in the database (e.g. due to chemotherapy-induced toxicity, disease progression, at patients request, other reasons). |
| Hospital admission | Unplanned treatment-related hospital admissions (days of hospitalization). |
